# Dynamic Regulation of Proteasome Expression

**DOI:** 10.3389/fmolb.2019.00030

**Published:** 2019-05-01

**Authors:** Ryo Motosugi, Shigeo Murata

**Affiliations:** Laboratory of Protein Metabolism, Graduate School of Pharmaceutical Sciences, The University of Tokyo, Tokyo, Japan

**Keywords:** proteasome, transcription, NRF1, immunoproteasome, thymoproteasome

## Abstract

The 26S proteasome is a multisubunit complex that catalyzes the degradation of ubiquitinated proteins. The proteasome comprises 33 distinct subunits, all of which are essential for its function and structure. Proteasomes are necessary for various biological processes in cells; therefore, precise regulation of proteasome expression and activity is essential for maintaining cellular health and function. Two decades of research revealed that transcription factors such as Rpn4 and Nrf1 control expression of proteasomes. In this review, we focus on the current understanding and recent findings on the mechanisms underlying the regulation of proteasome expression, as well as the translational regulation of proteasomes.

## Introduction

**Figure 1:**
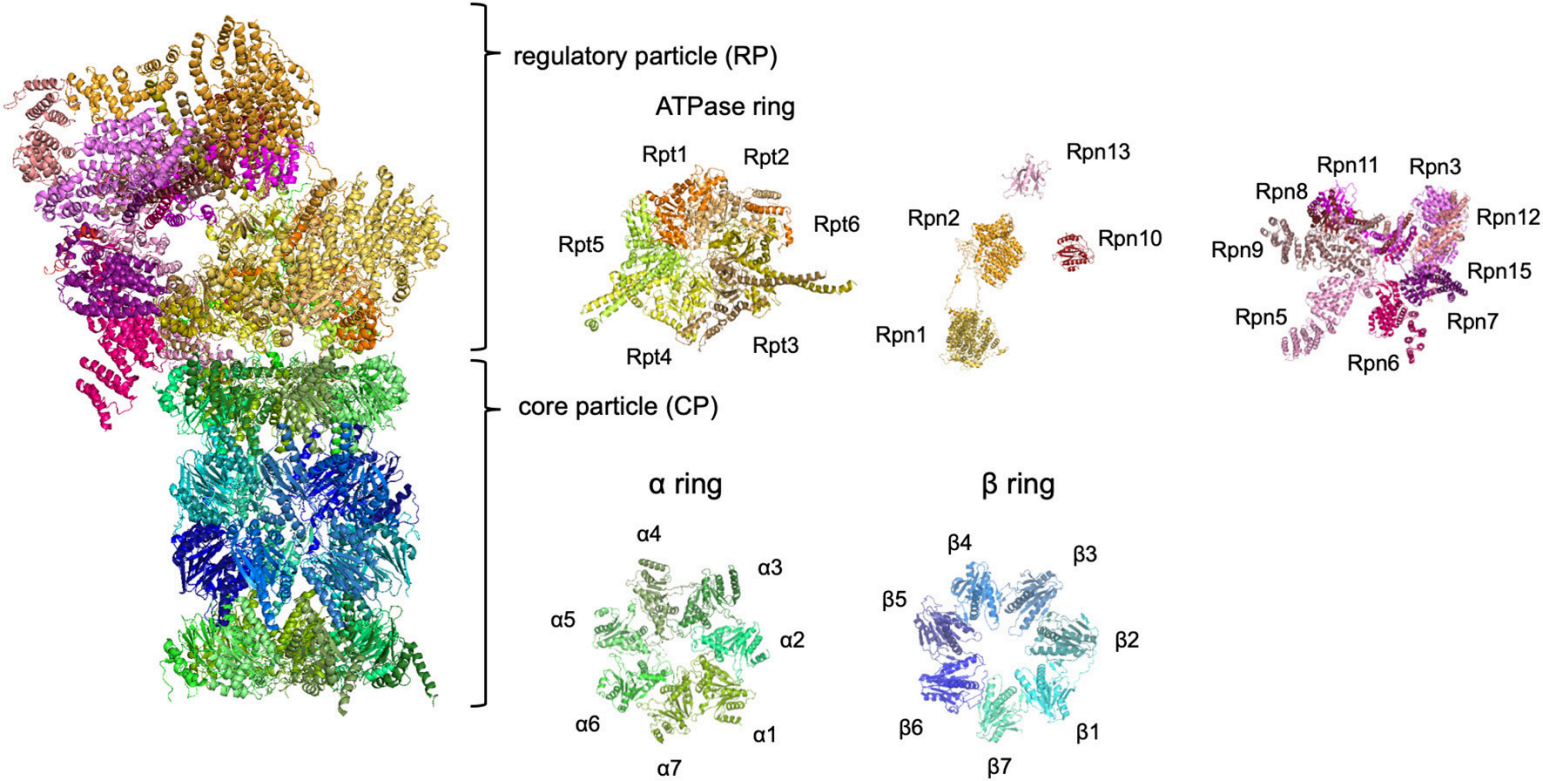
The architecture of the 26S proteasome. The human 26S proteasome structures obtained by cryo-electron microscopy. Because Rpn13 is not included in the cryo-EM data, the structural data of Rpn13 obtained by solution NMR was also used. The structural data were derived from RCSB PROTEIN DATA BANK (PDB ID: 6MSK and 5YMY).

The ubiquitin-proteasome system (UPS) is a major protein degradation pathway present in all eukaryotes. Substrate proteins are covalently modified by ubiquitin, recognized by ubiquitin receptors, and degraded by the proteasome. The 26S proteasome is a large protease complex that selectively recognizes, unfolds, and degrades ubiquitinated proteins in an ATP-dependent manner (Baumeister et al., 1998). The 26S proteasome consists of the 20S core particle (CP) and the 19S regulatory particle (RP) (Figure 1). The CP is a barrel-shaped complex composed of two heptameric α-rings (α1–α7) and two β-rings (β1–β7) arranged in an α-β-β-α order. Three catalytic subunits with protease activity (β1, β2, and β5) are contained within the β-ring. The RP comprises six AAA^+^ ATPase subunits (Rpt1–Rpt6) and 13 non-ATPase subunits (Rpn1–Rpn3, Rpn5–Rpn13, and Rpn15). The ATPase subunits form a hetero-hexameric structure that mediates substrate unfolding, CP gate opening, and translocation of substrates into the CP. Rpn1, Rpn10, and Rpn13, which contain a specific motif for recognition of ubiquitin or ubiquitin-like domains, bind to ubiquitin chains (Deveraux et al., 1994; Husnjak et al., 2008; Shi et al., 2016). Rpn11 acts as a deubiquitinating enzyme that catalyzes the removal of ubiquitin chains from substrates (Verma et al., 2002). Ubiquitin-specific protease 14 and ubiquitin carboxyl-terminal hydrolase 37 are deubiquitinating enzymes that interact with Rpn1 and Rpn13, respectively (Hamazaki et al., 2006; Shi et al., 2016). The complex structure of the proteasome requires precise assembly for the generation of a functional unit. The CP and RP are constructed separately with the assistance of specific assembly chaperones. During assembly of the CP, PAC1–PAC4/Pba1-Pba4 and POMP/Ump1 assist in the formation of the α-ring and β-ring in mammals/yeast (Bai et al., 2014). POMP/Ump1 also mediates dimerization of half-CPs to form mature CPs. Assembly of the RP is mediated by p27/Nas2, p28/Nas6, S5b/Hsm3, and PAAF/Rpn14.

Because the proteasome catalyzes the degradation of many proteins associated with various biological processes, maintaining adequate proteasome activity is essential for cellular homeostasis. Inappropriate increases or decreases in proteasome activity underlie various human diseases (Kumatori et al., 1990; Chen and Madura, 2005; Rubinsztein, 2006; Tomaru et al., 2012). Proteasome activity is decreased in autoinflammatory syndromes and neurodegenerative diseases but increased in cancer cells. To ensure precise modulation of proteasome activity, expression of proteasome subunits and activity of proteasome assembly chaperones needs to be controlled adequately. The abundance of proteasomes or proteasome subunits is associated with specific physiological features of cells, such as lifespan, senescence, and pluripotency. Increased expression of Rpn4, which acts as a transcription factor, extends the lifespan of budding yeast (Kruegel et al., 2011). In multicellular organisms such as aging fruit flies, overexpression of Rpn11 restores 26S proteasome activity, resulting in lifespan extension (Tonoki et al., 2009). Increased expression of Rpn6 extends the lifespan of nematodes under proteotoxic stress conditions (Vilchez et al., 2012a). In human embryonic stem cells, increased expression of Rpn6 maintains high levels of proteasome activity (Vilchez et al., 2012b). Overexpression of β5 restores proteasome activity in multipotent human bone marrow stromal cells, improves the senescent phenotype, and maintains pluripotency (Lu et al., 2014). Since these subunits are not supposed to work outside the proteasome, it is most likely that the increase in the proteasome activity is responsible for these effects. While further investigations are needed to clarify how overexpression of a single subunit recovers the proteasome activity, an increase or decrease in proteasome expression is involved in various biological processes. Here, we summarize the current understanding of the molecular mechanisms underlying the regulation of proteasome expression.

## Transcriptional Mechanisms Leading to Constitutive Proteasome Expression

Expression of proteasome subunits and proteasome assembly chaperones is coordinately regulated at the transcriptional level to maintain proteasome function. Although this regulatory mechanism remains to be elucidated, certain transcription factors involved in proteasome regulation were identified in the last two decades. In budding yeast, coordinated expression of proteasome genes is mediated by Rpn4. Rpn4, originally identified as a protein associated with the 26S proteasome, binds to the conserved sequence motif known as proteasome-associated control element (PACE) in the promoter region of all proteasome subunit genes and some proteasome assembly chaperone genes (Mannhaupt et al., 1999; Shirozu et al., 2015). The Rpn4 protein is extremely short-lived (t_1/2_ ≈ 2 min) and is degraded continually by the proteasome; therefore, Rpn4 accumulates and promotes proteasome expression under conditions of proteasomal dysfunction (Xie and Varshavsky, 2001), thereby playing a compensatory role in response to decreased proteasome activity. By contrast, lack of Rpn4 or PACE sequences in yeast decreases proteasome activity and resistance to various stresses such as DNA damage and oxidation (Wang et al., 2008). The transcription factor Rpn4 is therefore essential for stress-induced proteasome expression in a negative feedback loop, as well as for its constitutive expression.

Despite the importance of Rpn4 in yeast, there are no orthologs of Rpn4 or the PACE sequence in mammalian cells. However, several transcription factors that regulate the expression of proteasome subunits have been identified. Nuclear transcription factor Y (NF-Y) is a complex composed of NF-YA, NF-YB, and NF-YC; this complex binds to the CCAAT motif in the promoter of target genes. The CCAAT motif is present in six CP subunit genes (α2, α5, α7, β3, β4, and β6), five RP subunit genes (Rpt1, Rpt5, Rpt6, Rpn10, and Rpn11), and one assembly chaperone (p28) (Xu et al., 2012). Knockdown of NF-YA downregulates proteasome genes and decreases cellular proteasome activity. Forkhead box protein O4 (FOXO4), the mammalian ortholog of DAF-16 in nematodes, is crucial for maintenance of increased levels of Rpn6 and high proteasome activity in human embryonic stem cells (Vilchez et al., 2012b). Signal transducer and activator of transcription 3 (STAT3), which is activated by JAK phosphorylation upon cytokine signaling in the JAK/STAT pathway, regulates expression of β subunits and mediates epidermal growth factor-induced proteasome upregulation (Vangala et al., 2014). Knockdown of STAT3 downregulates β5; accumulation of activated STAT3 results in the induction of β5. These findings explain the induction of a particular set of constitutive proteasome subunits by specific transcription factors in mammalian cells; however, the significance of the mechanism by which different transcription factors separately regulate a part of the subunits is not understood. A recent study suggested that the compensatory increase in proteasome expression in mammalian cells is induced by nuclear factor erythroid-derived 2-related factor 1 (NFE2L1, also known as Nrf1) (Radhakrishnan et al., 2010). Nrf1 upregulates expression of all proteasome subunits and proteasome assembly chaperones in response to proteasome inhibition, leading to *de novo* proteasome synthesis. Therefore, Nrf1 is considered a pivotal regulator of proteasome expression in the presence of proteasomal dysfunction.

## Molecular Characteristics of the Transcription Factor Nrf1

Nrf1 belongs to the cap “n” collar basic leucine zipper (CNC-bZIP) family of transcription factors, which includes six transcription factors: Nrf1 (Chan et al., 1993), Nrf2 (Moi et al., 1994), Nrf3 (Kobayashi et al., 1999), nuclear factor erythroid 2 (NF-E2) p45 subunit (Andrews et al., 1993), BTB and CNC homolog 1 (Bach1), and Bach2 (Oyake et al., 1996). All of these proteins contain a CNC domain and a bZIP domain. The bZIP domain has two structural features, a region enriched in arginine and lysine residues (basic region) and a heptad repeats of leucine residues (leucine zipper) (Ellenberger, 1994). The basic region recognizes a specific DNA sequence, whereas the leucine zipper mediates dimerization with other bZIP proteins. Because CNC-bZIP proteins cannot bind DNA as monomers, they form heterodimers with small Maf proteins, which are also bZIP proteins, for transcriptional activation (Johnsen et al., 1996). The CNC domain is conserved between insects and mammals and is necessary for the DNA binding and transactivation capacity of CNC-bZIP proteins (Sykiotis and Bohmann, 2010).

A heterodimer of a CNC-bZIP protein and a small Maf protein binds to the antioxidant response element (ARE) sequence located in the promoter regions of various genes involved in antioxidant responses and metabolic regulation (Rushmore et al., 1991; Wasserman and Fahl, 1997). The ARE core sequence 5′-RTGACnnnGC-3′ corresponds to the Nrf1 binding consensus sequence 5′-RTGACTCAGC-3′, which was recently identified using chromatin immunoprecipitation (Baird et al., 2017). Notably, this binding sequence is present in the promoter region of all 33 proteasome subunit genes. Although Nrf2 recognizes the same consensus sequence as Nrf1, proteasome genes are predominantly regulated by Nrf1 and not Nrf2 (Hirotsu et al., 2012; Baird et al., 2017). However, Nrf2 induces proteasome expression in several types of cancer cells in a p53-dependent manner (Walerych et al., 2016). Although the role and target genes of Nrf1 differ from those of Nrf2, the precise mechanisms underlying the regulation of these two transcription factors need further investigation.

## Molecular Mechanism of Nrf1 Activation

**Figure 2:**
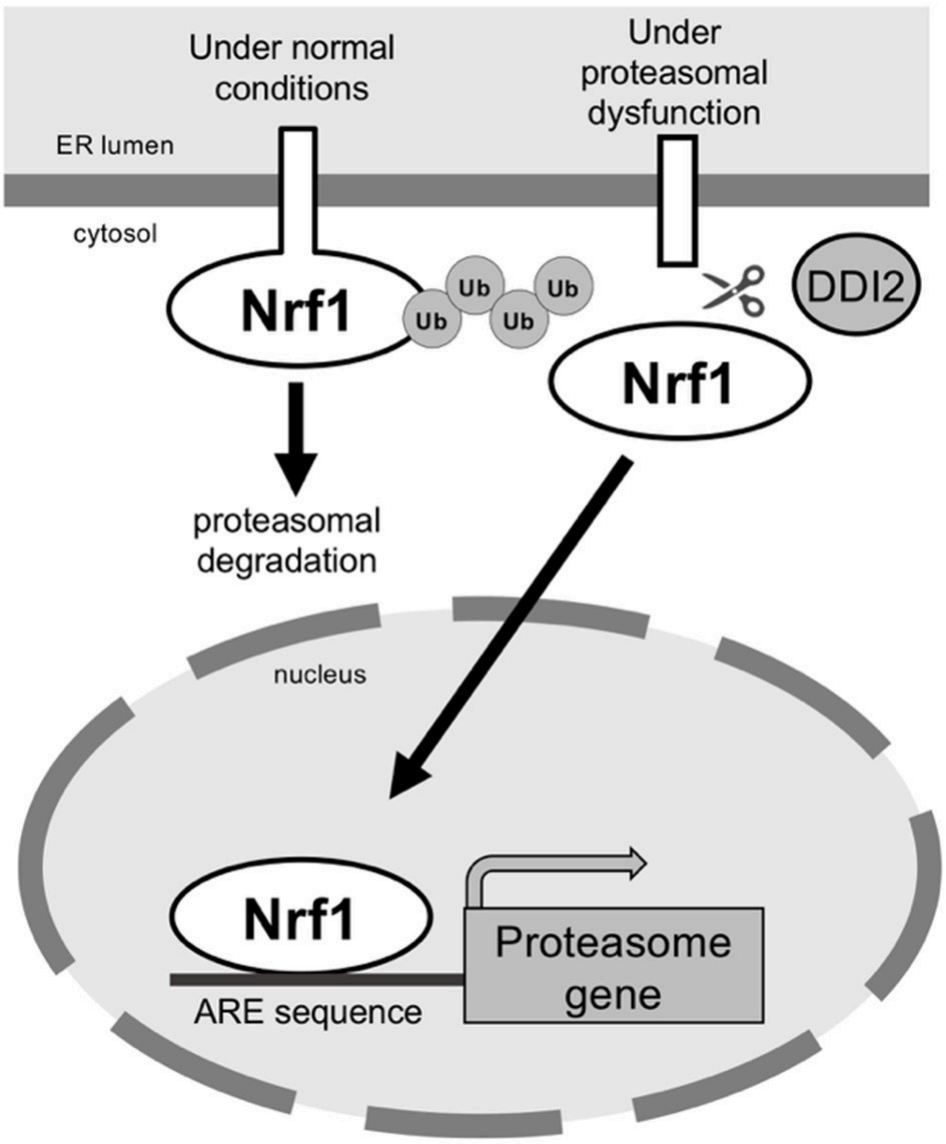
The transcription factor Nrf1 is processed and activated by DDI2 under conditions of proteasomal dysfunction. Under normal conditions, Nrf1 is degraded via the ERAD pathway. In proteasomal dysfunction, Nrf1 is processed by the aspartic protease DDI2; the mature form of Nrf1 then translocates to the nucleus to induce expression of proteasome genes.

Normally, Nrf1 localizes to the endoplasmic reticulum (ER) via a transmembrane domain in its N-terminus (Wang and Chan, 2006; Zhang et al., 2007). ER-associated protein degradation (ERAD) mediates degradation of misfolded ER-resident proteins, and Nrf1 is continuously degraded by the ERAD pathway. In this pathway, Nrf1 is translocated from the ER to the cytosol by the AAA^+^ ATPase p97/valosin containing protein, ubiquitinated, and degraded by the proteasome (Tsuchiya et al., 2011). Because Nrf1 undergoes rapid degradation by the proteasome, inhibition of the proteasome results in significant accumulation of Nrf1, which leads to compensatory expression of proteasome subunit genes (Figure 2).

Nrf1 is cleaved and translocated to the nucleus, where it becomes transcriptionally active (Radhakrishnan et al., 2014; Sha and Goldberg, 2014). DNA damaged inducible 1 homolog 2 (DDI2; DDI1/VSM1 in yeast), an aspartic protease, was recently identified as a critical regulator of Nrf1 activation (Koizumi et al., 2016). In DDI2 mutant cells, the processed form of Nrf1 disappears, and the full-length form accumulates. Moreover, compensatory expression of proteasome genes in response to proteasome inhibition is suppressed in DDI2-deficient cells. The contribution of DDI family proteins to Nrf1 activation was also observed in nematodes (Lehrbach and Ruvkun, 2016). These findings suggest that the mechanism underlying Nrf1 activation is highly conserved among multicellular organisms.

## Proteasome Expression in Response to Nutrient Conditions

**Figure 3:**
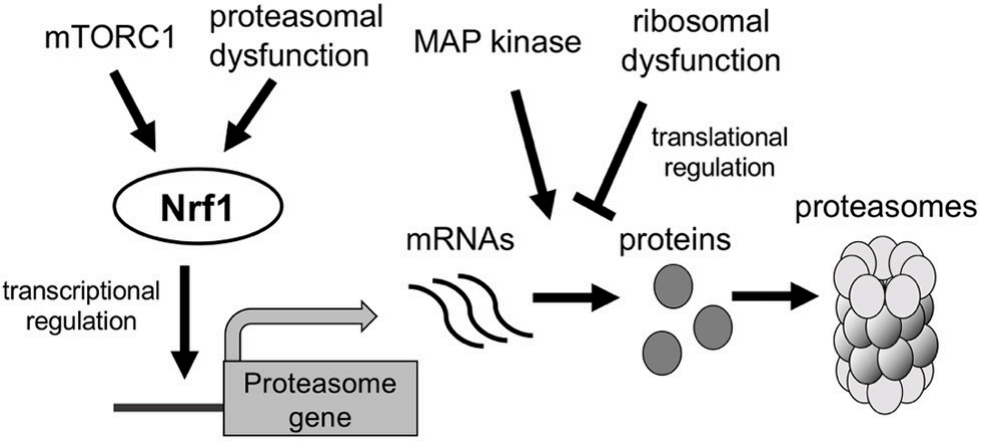
Regulation of the expression of proteasome genes. Proteasome dysfunction and mTORC1 activation induce transcription of proteasomal genes in an Nrf1-dependent manner. MAP kinase and ribosomal dysfunction are suggested to regulate translation of proteasomal mRNAs.

Mechanistic target of rapamycin complex 1 (mTORC1) is activated in the presence of high nutrient levels or in response to growth factors; it then promotes cell growth and proliferation by inducing protein and lipid synthesis (Dibble and Manning, 2013). Besides, mTORC1 activation promotes protein degradation by upregulating proteasome expression, thereby increasing the intracellular pool of amino acids for new protein synthesis (Zhang et al., 2015). Sterol regulatory element binding protein (SREBP-1), encoded by the *SREBF1* gene, regulates expression of lipogenic genes; its activation is stimulated by growth factor signaling through mTORC1 (Ricoult and Manning, 2013). Recent studies indicate that SREBP-1 is activated by mTORC1 and induces Nrf1 expression and thus proteasome expression (Figure 3) (Zhang and Manning, 2015; Zhang et al., 2015). However, activated mTORC1 phosphorylates and inhibits ATG proteins involved in autophagy induction, including ATG13 and ATG1 (Hosokawa et al., 2009; Dibble and Manning, 2013). Accordingly, mTORC1 oppositely regulates the UPS and autophagy in response to nutrient conditions.

PI3K/Akt signaling, a key regulator of mTORC1 activity, negatively regulates FOXO transcription factors, which induce cell cycle arrest and apoptosis (Brunet et al., 2010; Zhang et al., 2011). Although mTORC1 and FOXO transcription factors play different roles in cell growth and proliferation, both promote the expression of proteasome genes. These intriguing facts suggest that improving our understanding of proteins targeted for proteasomal degradation and identifying ubiquitin ligases that are activated by mTORC1 or FOXOs is essential for elucidating the physiological significance of proteasomal degradation for cell growth, proliferation, and death.

## Transcriptional Regulation of the Immunoproteasome

**Figure 4:**
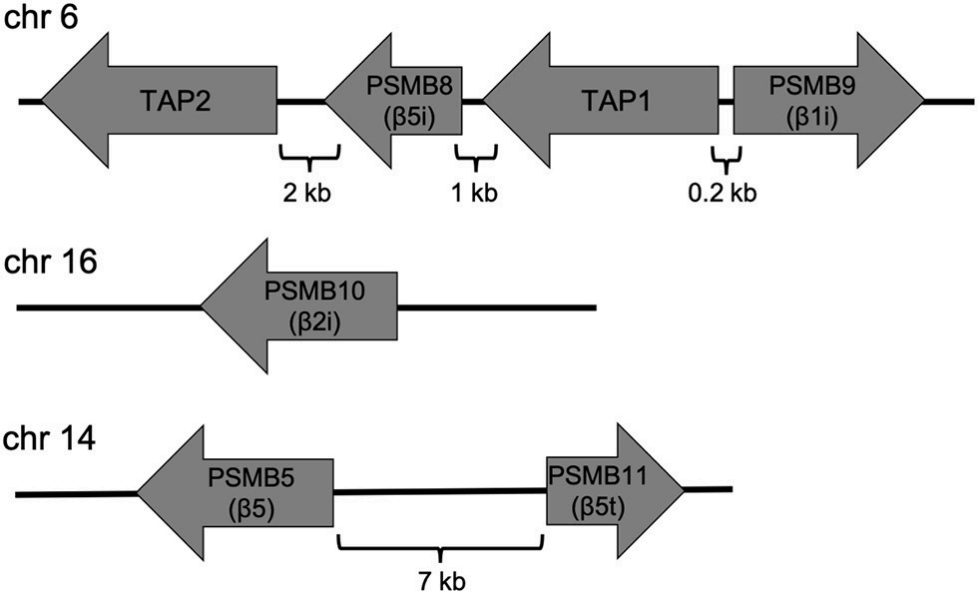
Genomic organization of the subunits of the immunoproteasome and thymoproteasome in humans. The genes encoding β1i (PSMB9) and β5i (PSMB5) are located in the MHC region and adjacent to the TAP1 and TAP2 genes. The β2i gene (PSMB10) is located outside the region. The gene encoding the thymoproteasome subunit β5t (PSMB11) is located close to the β5 gene (PSMB5).

Major histocompatibility complex (MHC) class I antigens are generated by the proteasome. Antigens are degraded by the proteasome, thereby generating small peptides that are translocated into the ER by the transporter associated with antigen processing (TAP), a heterodimer consisting of TAP1 and TAP2; these peptides then associate with MHC class I molecules (Leone et al., 2013). The immunoproteasome is a proteasome subtype that is induced by exposure to proinflammatory cytokines such as TNF-α and IFN-γ (Aki et al., 1994; Hallermalm et al., 2001). Immunoproteasomes have three catalytic subunits, namely β1i (LMP2), β2i (MECL-1), and β5i (LMP7), which correspond to β1, β2, and β5, respectively. These subunits catalyze the production of peptides that bind efficiently to MHC class I proteins (Kloetzel, 2001). Genes encoding β1i (*PSMB9*) and β5i (*PSMB8*) are located in the MHC class II region adjacent to the genes encoding TAP1 and TAP2, whereas the β2i gene (*PSMB10*) is located outside the MHC locus (Figure 4). β1i, β2i, and β5i are expressed in response to inflammatory stimuli and oxidative stress (Aki et al., 1994; Hallermalm et al., 2001; Hussong et al., 2010). IFN-γ induces expression of molecules associated with antigen presentation, including TAP1 and TAP2 (Ma et al., 1997), as well as the proteasome activator PA28αβ (Realini et al., 1994; Ahn et al., 1995). However, immune cells constitutively express immunoproteasomes at high levels. Hence, proteasomal subtypes are differentially expressed in cells according to cellular conditions and function.

The β1i and TAP1 genes share a bidirectional promoter characterized by the lack of a TATA box and the presence of several GC boxes (Wright et al., 1995). This region contains interferon consensus sequence 2 and γ-interferon-activated sequence sites, which interact with interferon regulatory factor 1 (IRF1) and STAT1 (Chatterjee-Kishore et al., 2000). IRF1 and STAT1 are mainly responsible for β1i induction in response to IFN-γ stimulation. The NF-κB transcription factor is necessary for TNF-α-dependent induction, and basal expression of β1i requires the GC box and the transcription factor Sp1 (Wright et al., 1995). Similar to β1i, the β5i promoter region contains a GC-rich region and NF-κB consensus sequence, along with a TATA-box (Zanelli et al., 1993). The transcription factor Zif268 (Egr1/Krox24/NGF-IA) induces expression of β1i and β5i in neuronal cells and regulates proteasome activity (James et al., 2006). The β2i gene is located outside the MHC region; however, its expression is also upregulated in response to IFN-γ (Hisamatsu et al., 1996). The promoter region of β2i does not contain CAAT or TATA boxes, whereas it contains Sp1, NF-κB, and IRF-1 binding sequences (Cruz et al., 1997; Hayashi et al., 1997). Hence, transcription of the β1i, β5i, and β2i genes is regulated by similar mechanisms.

## Transcriptional Regulation of the Thymoproteasome

Immature thymocytes undergo positive and negative selection in the thymic cortex and medulla, respectively. The thymoproteasome, the most recently identified proteasomal subtype, is expressed specifically in the thymic cortex and is essential for positive selection of CD8^+^ T cells (Murata et al., 2007, 2018). The β5t catalytic subunit is unique to thymoproteasomes and expressed exclusively in cortical thymic epithelial cells (cTECs), whereas immunoproteasomes are expressed in medullary thymic epithelial cells (mTECs). The gene encoding β5t (*PSMB11*) is adjacent to that for β5 (*PSMB5*) (Figure 4), and the gene product is encoded by a single exon in both the human and mouse genomes. A recent study showed that the forkhead transcription factor FOXN1, a master regulator of TEC lineage specification (Romano et al., 2013), promotes β5t expression directly (Uddin et al., 2017). The promoter region of the β5t gene contains the highly conserved Foxn1-binding core sequence 5′-ACGC-3′; a point mutation in this sequence decreases β5t expression and CD8^+^ T cell production. Despite the presence of the FOXN1-β5t transcriptional axis in cTECs, FOXN1 does not always induce β5t expression as FOXN1 is also expressed in mTECs, which do not express β5t at detectable levels. This suggests the existence of an unidentified cellular context unique to cTECs that is required for β5t expression.

## Translational Regulation of Proteasomes

A recent investigation of the proteasome demonstrated that proteasomal gene expression is regulated not only at the transcriptional level but also by a post-transcriptional or translational mechanism. In yeast, the mitogen-activated protein kinase Mpk1 maintains proteasome levels in response to tunicamycin and rapamycin, which upregulate expression of proteasome subunits and 19S regulatory particle assembly chaperones (RACs) in an Mpk1-dependent manner; however, mRNA levels do not differ between wild-type and Mpk1-deficient cells (Figure 3) (Rousseau and Bertolotti, 2016), suggesting that Mpk1 regulates expression of the proteasome and RACs under stress conditions at the translational level. Haploinsufficiency of ribosomal protein genes, which is the primary cause of Diamond-Blackfan anemia, suppresses translation of Rpt5 (encoded by *PSMC3*) mRNA (Khajuria et al., 2018). Therefore, the shortage of available ribosomes selectively inhibits translation of proteasomes in hematopoietic stem and progenitor cells, thereby impairing erythroid lineage commitment. These findings suggest that proteasome abundance is regulated at the post-transcriptional or translational level, although the underlying molecular mechanism remains unclear.

## Concluding Remarks

Many studies have attempted to elucidate the mechanisms underlying the regulation of proteasome expression, leading to the identification of several transcription factors. In yeast, Rpn4 acts as a transcription factor under normal and proteasome-impaired conditions. In mammals, Nrf1 promotes expression of all proteasome subunits in response to proteasomal dysfunction. Although specific transcription factors that regulate the expression of proteasome subunits under normal conditions have been identified, a universal mechanism regulating expression of all subunits remains to be elucidated. In addition, recent findings suggest that proteasome expression is regulated not only at the transcriptional level but also at the translational level; therefore, a comprehensive understanding of the molecular mechanisms underlying the regulation of proteasome expression remains elusive. Because precise regulation of proteasome expression is essential for cellular homeostasis, and its failure is associated with various diseases such as cancer, neurodegenerative disease, inflammatory, and immunological diseases, and senescence, further studies are warranted to elucidate the mechanism underlying the regulation of proteasome expression in a physiological and pathological context.

## Author Contributions

All authors listed have made a substantial, direct and intellectual contribution to the work, and approved it for publication.

### Conflict of Interest Statement

The authors declare that the research was conducted in the absence of any commercial or financial relationships that could be construed as a potential conflict of interest.
